# Prospective Assessment of Scoliosis-Related Anxiety and Impression of Trunk Deformity in Female Adolescents Under Brace Treatment

**DOI:** 10.1007/s10882-012-9296-y

**Published:** 2012-09-04

**Authors:** Maciej Glowacki, Ewa Misterska, Katarzyna Adamczyk, Joanna Latuszewska

**Affiliations:** 1 Department of Pediatric Orthopaedics and Traumatology, Poznan University of Medical Sciences, 61-545 Poznan, ul. 28 Czerwca 1956 135/147, Poland; 2 Department of Human Development Psychology and Family Studies, Adam Mickiewicz University, 60-568 Poznan, ul. Szamarzewskiego 89, Poznan, Poland; 3 Department of Motor System Rehabilitation, Poznan University of Physical Education, 61-871 Poznań, ul. Królowej Jadwigi 27/39, Poland

**Keywords:** Spinal deformity, Adolescence, Anxiety, Body appearance, SAQ, STAIC-trait

## Abstract

The aim of this study is to make a prospective analysis of changes in anxiety levels and determining their associations with a longitudinal subjective assessment of trunk deformity in adolescent females with scoliosis, in relation to clinical, radiological and brace-related data. The study design was comprised of three questionnaire assessments, with the second and third evaluations taking place 6 and 12 months after the beginning of the study, respectively. 36 AIS females treated conservatively were asked to fill in the Polish versions of the Spinal Appearance Questionnaire (SAQ-pl) and the trait version of the Spielberger’s Anxiety Inventory for Children (STAIC-trait). High anxiety was indicated in 16.6, 8.3 and 8.3% during the 1st, 2nd and 3rd evaluations. Patients’ results differ in regards to the Curve domain; the discrepancies concern the 2nd and 3rd and the 1st and 3rd evaluations (*p* = 0.028 and *p* = 0.003, respectively). The only association between STAIC-trait and SAQ-pl regards Trunk shift in the 1st evaluation (rs = 0.48). The logistic regression revealed that the duration of brace-wearing in months has a statistically significant (*p* = 0.021) influence on the probability of diagnosing patients’ low anxiety levels in the 2nd assessment. Special attention should be paid to patients’ emotional reactions later on as brace-wearing continues as well as to the results which support the point that patients’ perceptions of spinal deformity do not deteriorate with treatment time. Clinicians need to be aware how patients’ appearance-specific cognitions might be associated with levels of emotional distress and relate to clinical and radiological, scoliosis-related data.

## Introduction

Bracing in adolescent idiopathic scoliosis (AIS) has been shown to have favourable physical and structural outcomes, despite also being a very stressful or traumatic experience for some patients (Fernandez-Feliberti et al. 1995; Weiss 2003; Rivett et al. 2009). It is emphasized the combination of AIS as a disease and brace-wearing may disturb social interaction or mental health in general (Vasiliadis et al. 2006; Eliason and Richman 1984; Reichel and Schanz 2003) Moreover, since AIS affects trunk shape, it has the potential to adversely affect lifestyle, behavior, and self-esteem (Rivett et al. 2009). A negative self-evaluation of appearance may lead to different reactions such as body dissatisfaction, negative body image, concern with body size and shape (Dixit et al. 2011).

Several studies were related to different aspects of mood disorders in females with AIS, such as depression or anxiety (LaMontagne and Salisbury 2011; Hawes 2002; Payne et al. 1997; Clayson et al. 1981; Freidel 1999). However, most of the analyzed research focused on spinal surgery- and scoliosis-related anxiety. Anxiety and pain were considered to be major concerns not only for children who underwent surgery, but also for their parents and health care professionals (LaMontagne et al. 2001). Hawes (2002) indicated AIS-related anxiety has been shown to be the result of not knowing whether the spinal deformity and its symptoms will progress. Payne et al. (1997) showed that girls with scoliosis were 55% more likely to have suicidal thoughts, and three times more likely to consume alcohol after school than healthy controls. Clayson et al. (1981) preoperatively investigated adaptive personality characteristics such as trait anxiety, as predictors of postoperative physical and psychological patterns in scoliosis, whereas LaMontagne et al. (2001) pointed out that studying both parents and children helped to explain the variance in children’s self-reported anxiety.

Relevant literature offers data on the negative body image and its relationship with broad aspects of emotional and interpersonal functioning among adolescents (Davison and McCabe 2006). Furthermore, Choate provides strong evidence that body image dissatisfaction is linked with poor self-esteem, anxiety about social evaluation, public self-consciousness and depression (Choate 2005). Therefore, we believe the emotional state of AIS females may be related to patients’ concerns and perceptions of body appearance, since high anxiety often affects patients’ cognitive functions (Peretti 1998). Moreover, the complete evaluation of psychological well-being in the course of conservative treatment due to AIS should include a prospective analysis of changes in assessment of body disfigurement in relation to different aspects of stress, such as scoliosis and brace treatment—related anxiety. Thus, the aim of our research is to produce a prospective analysis of changes in anxiety levels and determining their associations with longitudinal subjective assessment of trunk deformity in adolescent females with scoliosis, in relation to clinical, radiological and brace-related data.

## Material and Methods

### Study Design

The study design was longitudinal and was comprised of three clinical and questionnaire assessments. Radiological evaluation was performed during the first and the third assessments only. The second and the third evaluations took place 6 and 12 months after the beginning of the study, respectively.

The assessment tools were administered during a routine patient visit. The investigator was available during this time, if study participants required explanation or clarification. All patients received detailed information on the aim of the study and were assured of anonymity, following which they gave their informed consent. The study was approved by the Bioethics Committee. The study group was comprised of 36 consecutively selected female patients with AIS treated conservatively with a Cheneau brace and all recruited from one centre.

The inclusion criteria were as follows: a minimum duration of Cheneau brace application of at least 12 h a day, a Cobb angle of 20–40°, 10–17 years of age. We excluded patient in whom other diseases leading to trunk deformity or serious medical conditions were diagnosed. The first evaluation of patients took place at a mean of 17.9 months SD 17.6, the second at 24.5 months SD 17.5 and then 30.1 months SD 17.6 at the final assessment after beginning Cheneau brace treatment. On average, patients wore the brace for 15.9 h SD 2.9 (first evaluation), 15.6 h SD 2.6 (second) and 15.2 h SD 2.2 (third and final evaluation) per day.

Patients’ age was 13.4 years SD 1.7 in the first assessment, 14.0 years SD 1.9 in the second and 14.4 years SD 1.7 in the last evaluation. The mean Cobb angle was 27.1° SD 5.1 in the first and 24.9° SD 9.1 in the final examination.

Table 1 shows detailed clinical and socio-demographic characteristics of scoliosis patients. 58% of patients had thoracic scoliosis, 33% had thoraco-lumbar scoliosis, and the remaining 9% of patients had lumbar scoliosis. Th8 was the apical vertebra in ten patients; Th9 in four patients; Th10 in two patients; Th11 in five patients; Th12 in five patients. L1 was the apical vertebra in seven patients; L2 in one, and L3 in two patients.

**Table 1:** Clinical and radiological characteristics of study participants

Parameters	Mean (SD)	Range (Min-Max)	Mean (SD)	Range (Min-Max)	Mean (SD)	Range (Min-Max)
	1st evaluation		2nd evaluation		3rd evaluation	
Body mass index	17.4 (1.7)	12.9–21.0	17.8 (1.9)	13.7–22.2	18.1 (1.9)	14.0–22.7
Age [years]	13.4 (1.7)	10–16	14.0 (1.9)	10–17	14.4 (1.7)	11–18
Cobb angle	27.1 (5.1)	20–38	–	–	24.9 (9.1)	5–48
Angle of trunk rotation^a^	8.3 (3.1)	3–15	7.8 (3.5)	1–15	6.9 (3.5)	2–15
Apical translation [cm]^b^	2.1 (0.9)	0.4–4.0	–	–	2.1 (1.0)	0.2–4.2
Brace [hours/day]	15.9 (2.9)	8–22	15.6 (2.6)	9–22	15.2 (2.2)	9–18
Brace [in months]	17.9 (17.6)	1–69	24.5 (17.5)	5–76	30.1 (17.6)	12–81

^a^Angle of trunk rotation as measured with Perdriolli’s inclinometer

^b^The degree of the apical translation of the central sacral vertical line (CSVL) according to the Harms Study Group

### Questionnaires

Polish versions of the Spinal Appearance Questionnaire (SAQ-pl) and the trait version of the State-Trait Anxiety Inventory for Children (STAIC-trait) were filled in by patients (Spielberger 1973; Sanders et al. 2007; Jaworowska 2005; Misterska et al. 2011; Roy-Beaudry et al. 2011). The Spinal Appearance Questionnaire (SAQ) was developed from the Walter Reed Visual Assessment Scale (WRVAS) to measure patients’ and their parents’ perception of several aspects of the appearance of their spinal deformity. The instrument is divided into 2 sections: the first relies on drawings demonstrating varying severity levels of several components of spinal deformity, and the second relies on textual questions rating dissatisfaction with other aspects of spinal deformity appearance. The SAQ for patients consists of 20 items, which form the following subscales: General, Curve, Prominence, Trunk shift, Waist, Shoulders, Kyphosis, Chest and Surgical scar. The items are scored from 1 to 5 points. The higher the score the worse the patients’ perceived their appearance. The presented study applies to patients treated conservatively only, therefore we omitted the Surgical scar subscale in the analyses (Sanders et al. 2007; Misterska et al. 2011).

The State-Trait Anxiety Inventory for Children is comprised of separate, self-report scales for measuring two distinct anxiety concepts: state anxiety (STAIC-state) and trait anxiety (STAIC-trait). Only the trait version was used in the presented research to objectify the more lasting manifestations of anxiety. This version has proven to be useful for identifying children with high levels of neurotic anxiety and in the selection of subjects for psychological experiments who differ in motivation or drive levels. Moreover, it serves to evaluate the immediate and long-term effectiveness of clinical treatment procedures designed to reduce neurotic anxiety in children (Spielberger 1973; Jaworowska 2005). The STAIC-trait contains 20 items in which the child/adolescent is asked to rate the frequency with which (s)he experiences anxiety symptoms such as “I am scared”, “I feel troubled”, and “I get a funny feeling in my stomach” using three-point scales: *1* indicates almost never, *2* sometimes, and *3* often. In the presented research the total trait anxiety score was calculated by summing the ratings for all items, then it was referred to Polish norms expressed as sten scores, appointed for age and gender (Jaworowska 2005).

### Statistical Analysis

In respect to statistical quantitative features, we determined mean, 95% confidence intervals, range and standard deviations. In respect to qualitative features, we gave the number of units that belong to described categories of a given feature and their respective percentages. Wilcoxon signed ranks tests were used to compare questionnaire results between the three assessment times. Spearman’s rank order correlation coefficients were used to evaluate correlations between quantitative variables. Correlations were defined as strong as >0.60, moderate 0.30–0.60 and weak <0.30 respectively (Hinkle et al. 1998).

We have used the logistic regression analysis to evaluate the influence of the socio-demographic, brace-related and radiological data and anxiety levels on the probability of achieving a “good result” in the SAQ questionnaire. Based on the lower and upper quartile distribution of the results, the SAQ-pl total scores were split into two categories: “good result” (from 16 to 32 points) and “poor result” (above 32 points).

The logistic regression analysis was used to evaluate the influence of the socio-demographic, brace-related and radiological data and perception of trunk deformity on the probability of achieving low anxiety levels, according to Polish norms appointed for age and gender (Jaworowska 2005). As the border level of statistical significance we adopted *p* = 0.05; test results with a p value exceeding this level were treated as insignificant. Statistical calculations were performed by means of Statistica software.

## Results

### Scores Distribution

In regards to the total results of the SAQ-pl, patients scored 30.05 SD 5.9 in the 1st assessment, 29.2 SD 4.7 in the 2nd and 29.2 SD 5.1 in the 3rd and final assessment. In the 1st evaluation, patients exhibit the most self-criticism in the following order: General, Waist, Shoulders, Chest, Curve, Trunk shift and Kyphosis. Prominence was the element that was assessed the least critically by patients. In the 2nd assessment, patients exhibit the most criticism in the following order: General, Waist, Chest, Shoulders, Curve. Trunk shift, Kyphosis and Prominence were the least criticized elements of body shape in patients by the patients.

The most criticized elements of body shape in the 3rd evaluation were the following: General, Waist, Shoulders, Chest, Curve, Kyphosis, Trunk shift and Prominence (see Table 2).

**Table 2:** Distribution of results of SAQ-pl and STAIC-trait

	Mean (SD)	95% Confidence interval (from-to)	Range (Min-Max)	Mean (SD)	95% Confidence interval (from-to)	Range (Min-Max)	Mean (SD)	95% Confidence interval (from-to)	Range (Min-Max)
	1st evaluation			2nd evaluation			3rd evaluation		
SAQ-pl									
General	3.5 (0.7)	3.3–3.8	2,.0–4.3	3.5 (0.7)	3.2–3.7	1.7–4.7	3.5 (0.7)	3.3–3.8	1.7–5.0
Curve	2.4 (0.7)	2.2–2.7	1.0–4.0	2.1 (0.5)	2.0–2.3	1.0–4.0	2.0 (0.4)	1.8–2.1	1.0–3.0
Prominence	1.8 (0.4)	1.6–1.9	1.0–2.5	1.6 (0.5)	1.5–1.8	1.0–3.0	1.7 (0.5)	1.5–1.9	1.0–3.5
Trunk shift	1.9 (0.5)	1.7–2.0	1.0–3.0	1.8 (0.5)	1.6–1.9	1.0–2.5	1.8 (0.4)	1.6–1.9	1.0–2.5
Waist	3.4 (1.2)	3.0–3.8	1.0–5.0	3.2 (1.2)	2.8–3.6	1.0–5.0	3.0 (1.3)	2.6–3.5	1.0–5.0
Shoulders	2.9 (1.0)	2.5–3.2	1.0–5.5	2.7 (0.9)	2.4–3.0	1.0–4.0	2.7 (0.8)	2.4–3.0	1.0–4.0
Kyphosis	1.9 (0.6)	1.7–2.1	1.0–3.0	1.8 (0.7)	1.6–2.1	1.0–4.0	1.9 (0.5)	1.7–2.0	1.0–4.0
Chest	2.7 (1.5)	2.7–3.2	1.0–5.0	2.8 (1.6)	2.3–3.7	1.0–5.0	2.6 (1.5)	2.1–3.2	1.0–5.0
Total score	2.7 (0.6)	2.5–2.9	1.5–3.8	2.6 (0.5)	2.4–2.8	1.6–3.7	2.6 (0.5)	2.4–2.8	1.6–3.6
STAIC-trait									
Total score	30.0 (5.9)	28.0–32.0	22.0–45.0	29.2 (4.7)	27.6–30.8	20.0–41.0	29.2 (5.1)	27.5–30.9	20.0–40.0

SAQ-pl-Polish versions of the Spinal Appearance Questionnaire, STAIC-trait- State-Trait Anxiety Inventory for Children- the trait version

From the interpretation of the answers given to question 8, it seems that the head-chest-hips line (27.7%) and shoulder level line (27.8%) are the most disturbing issues for the majority of AIS patients in the 1st evaluation. Similarly, the head-chest-hips line is a matter of special concern for 33% of patients in the 2nd evaluation, whereas 27.8% of AIS females rated rib prominence as the most disturbing issue in the last evaluation.

Considering the answers given to question 18 applying to the most important question of items 9–12, it seems that for as many as 52.8% of patients in the first evaluation, 52.8% in the second and 55.6% in the last evaluation, the most important item is question 9 about the desire “to be more even”. The distribution of answers to question 20 concerning patients’ expectations regarding improvement of body shape, revealed that as many as 47.2% of patients in the 1st assessment would change their body shape in general. In the 2nd assessment the area of greatest concern was waist asymmetry and 33.3% would rather have a more symmetrical waist. In the last evaluation as many, as 30.5% of patients would rather have a  “straighter spine”.

In regards to the general results of STAIC-trait, patients scored 30.0 SD 5.9, 29.2 SD 4.7 and 29.2 SD 5.1 in the 1st, 2nd and 3rd assessments, respectively (see Table 2).

Table 3 presents the results of the interpretation of STAIC-trait total scores by referring individual patient’s results to Polish norms appointed for age and gender [18]. During the 1st evaluation most patients (83.4%) experienced low or medium anxiety, whereas after 6 and then after 12 months medium or low anxiety level was reported by 91.7% of AIS females in both periods (see Table 3). High anxiety was indicated in 16.6, 8.3 and 8.3% of study participants during the 1st, the 2nd and 3rd evaluations.

**Table 3:** Interpretation of STAIC-trait total scores according to Polish norms appointed for age and gender

Score interpretation	STAIC-trait % (n)		
	1st evaluation	2nd evaluation	3rd evaluation
Low anxiety	41.7 (15)	41.7 (15)	52.8 (19)
Medium anxiety	41.7 (15)	50.0 (18)	38.9 (14)
High anxiety	16.6 (6)	8.3 (3)	8.3 (3)

SAQ-pl-Polish versions of the Spinal Appearance Questionnaire,

STAIC-trait- State-Trait Anxiety Inventory for Children- the trait version

### Comparative Analyses

Patients’ results only differ in regards to the Curve domain in the SAQ-pl. The reported discrepancies concern the 2nd and 3rd evaluations and the 1st and 3rd evaluations (*p* = 0.028 and *p* = 0.003, respectively; the higher results regard the 1st and the 2nd evaluation) (Table 4).

**Table 4:** Results of comparisons between the 1st, 2nd and 3rd SAQ-pl and STAIC-trait results

SAQ-pl									STAIC-trait			
General	Curve	Prominence	Trunk shift	Waist	Shoulders	Kyphosis	Chest	Total score	STAIC-trait total score	Low anxiety	Medium anxiety	High anxiety
Comparison 1st evaluation - 2nd evaluation												
*p* = 0.509	*p* = 0.066	*p* = 0.223	*p* = 0.102	*p* = 0.244	*p* = 0.432	*p* = 0.650	*p* = 0.534	*p* = 0.257	*p* = 0.632	*p* = 1.000	*p* = 0.480	*p* = 0.289
Comparison 2nd evaluation - 3rd evaluation												
*p* = 0.841	*p* = 0.028*	*p* = 0.407	*p* = 1.000	*p* = 0.363	*p* = 0.574	*p* = 0.767	*p* = 0.323	*p* = 0.336	*p* = 0.732	*p* = 0.348	*p* = 0.346	*p* = 1.000
Comparison 1st evaluation - 3rd evaluation												
*p* = 0.762	*p* = 0.003*	*p* = 0.560	*p* = 0.153	*p* = 0.094	*p* = 0.305	*p* = 0.790	*p* = 0.872	*p* = 0.144	*p* = 0.537	*p* = 0.348	*p* = 0.811	*p* = 0.289

*p < 0.05, SAQ-pl-Polish versions of the Spinal Appearance Questionnaire, STAIC-trait- State-Trait Anxiety Inventory for Children- the trait version

### Correlational Analyses

Having analyzed the correlation between patient characteristics and SAQ-pl results in the 1st evaluation, we indicated a significant, but weak correlation between: assessment of Prominence and BMI (rs = −0.36) and apical translation (rs = 0.37). Anxiety level, as measured by STAIC-trait was related to apical translation only (rs = 0.41) (Table 5). In the 2nd evaluation the indicated weak correlations regarded perception of Prominence and Trunk shift in relation to angle of trunk rotation (rs = 0.33 and rs = 0.39, respectively). STAIC-trait was associated with duration of brace-wearing in months (rs = 0.34) (Table 6). Having analyzed the associations between the SAQ-pl and patient-related data in the 3rd assessment, we indicated a significant, although weak, correlation between patient General assessment of trunk shape and apical translation only (rs = 0.33). Anxiety levels were related, similarly as during the 2nd evaluation, to the monthly duration of orthosis wearing (rs = 0.33) (Table 7).

**Table 5:** Correlation between SAQ-pl patient and STAIC-trait results and patient characteristics during the 1st assessment

SAQ-pl patient form	General	Curve	Prominence	Trunk shift	Waist	Shoulders	Kyphosis	Chest	Total score	STAIC-trait
Body mass index	rs = −0.01	rs = −0.03	rs = −0.36*	rs = 0.01	rs = 0.06	rs = −0.18	rs = −0.26	rs = −0.34*	rs = −0.16	rs = −0.05
Age	rs = −0.16	rs = 0.12	rs = 0.24	rs = 0.06	rs = −0.15	rs = 0.13	rs = −0.02	rs = −0.11	rs = −0.06	rs = 0.23
Cobb angle	rs = −0.15	rs = 0.27	rs = 0.20	rs = 0.18	rs = −0.25	rs = −0.05	rs = 0.17	rs = −0.21	rs = −0.10	rs = 0.09
Angle of trunk rotation**	rs = −0.10	rs = 0.23	rs = 0.18	rs = 0.19	rs = −0.16	rs = 0.27	rs = 0.09	rs = 0.12	rs = 0.10	rs = −0.10
Apical translation***	rs = 0.17	rs = 0.11	rs = 0.37*	rs = 0.33	rs = 0.10	rs = 0.05	rs = 0.25	rs = 0.04	rs = 0.24	rs = 0.41*
Brace [hours/day]	rs = −0.09	rs = 0.06	rs = 0.31	rs = 0.06	rs = −0.07	rs = 0.08	rs = −0.04	rs = 0.19	rs = 0.07	rs = 0.10
Brace [in months]	rs = −0.06	rs = −0.14	rs = −0.09	rs = 0.05	rs = 0.01	rs = −0.04	rs = −0.05	rs = −0.17	rs = −0.14	rs = 0.02

*p < 0.05 **Angle of trunk rotation as measured with Perdriolli’s inclinometer ***The degree of the apical translation of the central sacral vertical line (CSVL) according to the Harms Study Group, SAQ-pl-Polish versions of the Spinal Appearance Questionnaire, STAIC-trait- State-Trait Anxiety Inventory for Children- the trait version

**Table 6:** Correlation between SAQ-pl patient and STAIC-trait results and patient characteristics during the 2nd assessment

SAQ-pl patient form	General	Curve	Prominence	Trunk shift	Waist	Shoulders	Kyphosis	Chest	Total score	STAIC-trait
Body mass index	rs = −0.09	rs = 0.28	rs = 0.18	rs = 0.11	rs = −0.02	rs = −0.06	rs = 0.03	rs = −0.27	rs = −0.09	rs = 0.24
Age	rs = 0.01	rs = 0.09	rs = 0.01	rs = 0.04	rs = 0.22	rs = 0.14	rs = −0.08	rs = −0.17	rs = −0.01	rs = 0.18
Angle of trunk rotation **	rs = −0.18	rs = 0.17	rs = 0.29	rs = 0.33*	rs = −0.20	rs = 0.18	rs = 0.39*	rs = 0.13	rs = 0.12	rs = −0.11
Brace [hours/day]	rs = −0.06	rs = 0.11	rs = 0.13	rs = −0.01	rs = 0.04	rs = −0.09	rs = −0.08	rs = 0.08	rs = 0.04	rs = −0.02
Brace [in months]	rs = −0.02	rs = −0.16	rs = 0.02	rs = 0.31	rs = −0.24	rs = 0,02	rs = 0.10	rs = −0.10	rs = −0.06	rs = 0.34*

*p < 0.05 **Angle of trunk rotation as measured with Perdriolli’s inclinometer, SAQ-pl-Polish versions of the Spinal Appearance Questionnaire, STAIC-trait- State-Trait Anxiety Inventory for Children- the trait version

**Table 7:** Correlation between SAQ-pl patient and STAIC-trait results and patient characteristics during the 3rd assessment

SAQ-pl patient form	General	Curve	Prominence	Trunk shift	Waist	Shoulders	Kyphosis	Chest	Total score	STAIC-trait
Body mass index	rs = −0.08	rs = 0.12	rs = 0.04	rs = 0.19	rs = 0.10	rs = −0.01	rs = −0.20	rs = −0.23	rs = −0.06	rs = 0.19
Age	rs = 0.09	rs = 0.02	rs = −0.20	rs = 0.17	rs = 0.20	rs = 0.01	rs = −0.31	rs = −0.06	rs = 0.01	rs = 0.12
Cobb angle	rs = 0.19	rs = 0.13	rs = 0.02	rs = 0.01	rs = −0.09	rs = 0.09	rs = 0.05	rs = 0.06	rs = 0.08	rs = 0.16
Angle of trunk rotation**	rs = −0.07	rs = 0.23	rs = 0.22	rs = 0.28	rs = −0.30	rs = 0.16	rs = 0.26	rs = 0.03	rs = −0.04	rs = 0.17
Apical translation***	rs = 0.33*	rs = 0.05	rs = −0.08	rs = −0.17	rs = 0.21	rs = 0.16	rs = −0.14	rs = 0.18	rs = 0.24	rs = 0.32
Brace [hours/day]	rs = 0.14	rs = 0.04	rs = −0.03	rs = 0.03	rs = 0.05	rs = 0.10	rs = 0.05	rs = 0.15	rs = 0.12	rs = −0.32
Brace [in months]	rs = 0.12	rs = −0.07	rs = 0.21	rs = 0.14	rs = 0.17	rs = 0.33	rs = −0.06	rs = 0.09	rs = 0.20	rs = 0.33*

*p < 0.05 **Angle of trunk rotation as measured with Perdriolli’s inclinometer ***The degree of the apical translation of the central sacral vertical line (CSVL) according to the Harms Study Group, SAQ-pl-Polish versions of the Spinal Appearance Questionnaire, STAIC-trait- State-Trait Anxiety Inventory for Children- the trait version

We performed a longitudinal analysis of the relations between perception of spinal appearance and anxiety level and indicated a significant moderate association between the STAIC-trait and assessment of Trunk shift only in the 1st evaluation (rs = 0.48) (Table 8).

**Table 8:** Correlation between SAQ-pl and STAIC-trait results

SAQ-pl patient form	General	Curve	Prominence	Trunk shift	Waist	Shoulders	Kyphosis	Chest	Total score
1st evaluation									
STAIC-trait	rs = 0.23	rs = −0.11	rs = 0.14	rs = 0.48*	rs = 0.19	rs = −0.12	rs = 0.07	rs = 0.06	rs = 0.19
2nd evaluation									
STAIC-trait	rs = 0.09	rs = −0.03	rs = 0.03	rs = −0.01	rs = 0.06	rs = −0.05	rs = −0.19	rs = −0.01	rs = 0.03
3rd evaluation									
STAIC-trait	rs = 0.08	rs = −0.29	rs = −0.15	rs = 0.04	rs = 0.16	rs = 0.20	rs = 0.06	rs = 0.11	rs = 0.11

*p < 0.05, SAQ-pl-Polish versions of the Spinal Appearance Questionnaire, STAIC-trait- State-Trait Anxiety Inventory for Children- the trait version

### Regression Analysis

The logistic regression analysis which aimed to evaluate the influence of the socio-demographic, brace-related and radiological data and anxiety level on the probability of achieving a “good result” in the SAQ questionnaire could not be performed due to the small number of scoliosis patients scoring a “good result” in the total score of SAQ-patient form in all 3 evaluations.

The logistic regression model obtained as a result of the calculations revealed that age, BMI, Cobb angle, angle of trunk rotation, apical translation, duration of brace wearing, or perception of spinal appearance in regards to SAQ-pl individual domains and the total score, do not have a statistically significant influence on the probability of patients’ achieving a low level of anxiety in the STAIC-trait, during the 1st and 3rd evaluation.

Interestingly, in the 2nd assessment, the logistic regression model revealed that duration of brace-wearing in months has a statistically significant (*p* = 0.021) influence on the probability of diagnosing a low anxiety level in patients. If brace-wearing duration increases by 1 month the probability of a patient reporting a low level of anxiety falls by 8%.

## Discussion

The purpose of the present study was to clarify changes in scoliosis-related anxiety and impression of spinal deformity and to account for prospective relationships between the psychological states and radiological and clinical data. It must be emphasized that little is known about changes in AIS patients’ assessment of spinal appearance across a longer time-frame perspective, as well as about relations between anxiety and cognitive functions or perceptions of spinal appearance in particular. Ascani et al. (1986) examined untreated idiopathic scoliosis patients, seen from a minimum of 15 to a maximum of 47 years after the end of growth and indicated the cosmetic appearance of these patients at long-term follow-up was better compared with that at the end of growth, even though the curves progressed. Edgar and Mehta (1988) examined how doctors perceived patients’ trunk shape in longer post-operative follow-up, reporting surgery improved spinal appearance; analysis of patients’ perspective revealed, however, they were not completely satisfied with the cosmetic result.

Noonan et al. (1997) found that the perception of self-image deteriorates with duration of follow-up, however this association was especially distinct in patients treated operatively as compared to patients treated conservatively. Meanwhile in the present study, regarding conservatively-treated patients only, it emerged that the participants’ perceptions of body shape and anxiety level do not deteriorate with time. Futhermore, the analyzed differences regarding SAQ results only, indicated improvement in the evaluation of body Curve, particularly after a 12 month observation treatment period.

Olafsson et al. (1995) evaluated the effect of brace treatment on five aspects of self-image: body-image, self-perception of skills and talents, emotional well-being, relations with family, and relations with others in patients with AIS and indicated there were no statistically significant differences between the answers of the scoliosis patients in the pre-bracing and follow-up interviews. Olafsson et al. (1995) concluded that, similarly to the present study, wearing a brace does not negatively affect the self-image of adolescents with idiopathic scoliosis.

Conservative or surgical scoliosis treatment can be regarded by young patients as a difficult, stressful and threatening situation and individuals may react with feelings of tension or anxiety (Reichel and Schanz 2003). At the same time, some constant features of human behaviour may predispose individuals to perceive certain situations as being dangerous or threatening, for example, when adolescents are being evaluated by others (Jaworowska 2005). Several studies have investigated depression or anxiety levels in AIS females (LaMontagne and Salisbury 2011; Hawes 2002; Payne et al. 1997; Clayson et al. 1981; Freidel 1999). As mentioned above, most of the analyzed research focused on spinal surgery- and scoliosis-related anxiety. In a study by LaMontagne et al. (2011), anxiety and pain were considered to be major concerns for scoliosis patients treated operatively, whereas Hawes (2002) indicated AIS-related anxiety has been shown to be the result of feelings of insecurity about whether the spinal deformity will progress. Results of the present study indicated that most of the AIS patients reported low and medium anxiety levels during the whole, observed treatment period of 12 months. Interestingly, the largest group of patients reporting high anxiety was recorded at the 1st assessment—16.6% of study participants, there were no confirmed significant differences between the amount of patients with high anxiety diagnosed at the 2nd and then at the 3rd assessment; which led to indicate that brace-related anxiety is constant throughout the course of orthosis treatment.

The pubertal changes and increase in body weight observed in adolescence may often result, especially among females, in intense focus being placed on the body and higher body dissatisfaction (Koff and Rierdan 1991). Therefore, our study was aimed, in addition, to investigate longitudinally during assumed a 12-month observation, which spine deforming factor affects AIS females’ perception of body appearance most adversely. Until now, many studies analyzed the ways deformities might be perceived by AIS patients as well as which scoliosis-related elements of body shape are the least acceptable (Sanders et al. 2003, 2007; Bago et al. 2007; Pratt et al. 2002; White et al. 1999). The quantitative data gained in our study revealed that General evaluation, Waist, Shoulders and Chest were the most criticized elements of body shape by patients, whereas Prominence was the least critically assessed element of trunk appearance in all evaluations. However, the interpretation of SAQ-pl qualitative data revealed that the head-chest-hips line and shoulder level constitute the most disturbing issues of scoliosis at the beginning of the study. Similarly, the head-chest-hips line was a matter of special concern for the largest amount of patients after the 6 months observation treatment period, whereas rib prominence, interestingly, was the most disturbing issue for almost one third of AIS females in the last evaluation.

The link between radiological and clinical assessment of scoliosis performed by physicians and patients’ perception of spinal disfigurement has been extensively studied, with the majority of investigations regarding operatively-treated patients (Sanders et al. 2007; White et al. 1999; Sadeghi et al. 2008; Haher et al. 1999). To date, many studies have shown no or weak relationships between size or severity of disfigurement and emotional distress, therefore assumptions that patients with extensive disfiguration have more psychological dysfunction than those with minor differences often have no empirical support (Robinson 1997). Following studies by D’Andrea et al. (2000), regarding operatively treated AIS patients, it was determined that with the exception of the Cobb angle, other radiological values do not correlate with self-image and the degree of deformity as assessed by patients in post-operative treatment. Similarly, Smith et al. (2006) found that there is little connection between radiological assessments and the level of satisfaction of patients. She found that a correlation exists with only post-operative values of the Cobb angle, the degree of post-operative correction, and the value of hump degree.

Interestingly, in a study by Sanders et al. (2007) it was ascertained that results achieved in respective subscales of the Spinal Appearance Questionnaire correlate with clinical and radiological aspects of deformities prior to surgical intervention. Pratt et al. concluded that the larger the deformity in the thoracic spine, the more critically the patients’ perceived their rib hump or hip and waist asymmetry. Similarly, he found that the higher the value of the Cobb angle in the thoracic-lumbar spine, the worse the perception of waist, hip and shoulder asymmetry was (Pratt et al. 2002). Moreover, in another study regarding SAQ, Misterska et al. (2011) found that only the value of the thoracic apical translation measured 2 years post-operatively was related to a lower probability of achieving a “good” general result in the Spinal Appearance Questionnaire.

Although it is very common to encounter discrepancies between the radiological or clinical evaluations of AIS and the patient perceptions of trunk appearance, the present study has indicated associations between the apical translation and assessment of Prominence as well as anxiety level regarding the beginning of the study. After 6 months the higher the value of the trunk rotation, the worse the assessment of prominence and trunk shift, additionally as the values of apical translation increased so did patients’ critical evaluation of General aspects of trunk shape after a 12-month observation period. This led us to support the above mentioned results regarding feasible associations between the objective evaluation of spinal deformity and patients’ subjective, nonverbal evaluation of body shape as applied in the SAQ.

Essau et al. (2000) found that adolescent females between the ages of 12 and 17 exhibit more anxiety symptoms than males and that this symptom tends to increase with age. Moreover, it was indicated that clinically significant body image concerns or a body image disorder are associated with higher levels of depression, anxiety or mood disorders in general (Dyl et al. 2006; Ferguson et al. 2011). In addition, dissatisfaction with body image may play a significant role in the development of low self-esteem, emotional problems and depression (Mori et al. 2009). Therefore, we assumed the emotional state, especially anxiety and tension, may be related to patients’ assessment of spinal appearance. Our study results support the evidence of relationships between patients’ perception of Trunk shift and anxiety, which indicated AIS patients’ subjective evaluation of body appearance might be influenced by their emotional states; however, the reported dependencies took place at the beginning of observation treatment period only.

The effect of brace use on the physical, psychological and social health of adolescent patients remains controversial and unambiguous. Differences in results may be derived from the type of brace applied, as well as differences in culture and the attitudes of patients (Cheung et al. 2007). Fallstrom et al. (1986) and Maclean et al. (1989) suggested that bracing had a psychological impact, causing low self-esteem and a more negative self-image, although there were no psychopathological changes in the long term. Cheung et al. (2007) indicated that bracing had a greater negative impact on the quality of life, especially on a function or activity level in patients with mild curves. Meanwhile, our results obtained during a 12-month observation period in the course of brace treatment do not support the discrepancies in regards to frequencies of analyzed anxiety levels. Interestingly, in the 2nd and the 3rd assessments, higher anxiety levels were related to an increase in the duration of brace treatment in months. The presented associations were supported by the regression analysis, showing the influence of even a single month extension in brace wearing on the probability of increasing patient anxiety.

Additionally, there were differences between the perception of body curve in the 2nd and 3rd evaluations as well as the 1st and 3rd evaluations, with a confirmed improvement in patient assessments of spinal appearance in this particular area. As far as the remaining SAQ-pl domains are concerned, we did not confirm any deterioration or improvement in patient functioning, considering the assumed points of the evaluation.

LaMontagne et al. (2011) investigated the relationships among children’s and parents’ preoperative and postoperative anxiety and children’s postoperative pain by means, as in the present study, of the Spielberger’s State-Anxiety scales and a visual analog scale. The authors indicated that children’s anxiety increased from preoperative to postoperative levels, and their postoperative anxiety levels positively related to their pain intensities on days 2 and 4 following the operation. Meanwhile, our study indicated constant anxiety levels during the evaluated 12-month treatment period. Moreover, our study results evidenced improvement of patients’ perception of body curve between the 1st to the last assessment as well as between the 2nd and the 3rd evaluation.

There are some limitations in this study that should be mentioned. We believe more a comprehensive evaluation of brace treatment should include longitudinally analyzed assessment of body shape from the perspective of male patients and both parents. Since little is known regarding the relationships between anxiety and depression disorders and cognitive functioning in scoliosis patient population, we postulate extending the knowledge available of associations between trunk appearance-specific cognitions and patients’ emotional states by means of quantitative and qualitative studies such as the Goodenough-Harris *Draw-A-Person Test* (Laak et al. 2005). Considering additional future research implications, it would be interesting to continue the presented research in regards to investigated domains of functioning across a longer follow-up after brace treatment.

LaMontagne et al. (2011) indicated information plus coping was most effective for reducing postoperative anxiety in adolescents with high preoperative anxiety. Therefore, considering the practical implications derived from the present study, we believe psychological support for AIS patients in the form of group or individual sessions should clearly be considered for inclusion in the entire course of treatment, since their positive influence in the prevention of psychosocial impairment has been confirmed (Reichel and Schanz 2003).

In conclusion, we believe physicians should pay special attention to patients’ emotional reactions later on as treatment with a brace progresses as well as to the results supporting the point that patient perceptions of spinal deformity do not deteriorate with treatment time. We postulate clinicians need to be aware that patients’ appearance-specific cognitions might be associated with levels of emotional distress, and how these factors relate to clinical and radiological, scoliosis-related data.
